# Identification of Tumor Antigens and Immune Subtypes of Glioblastoma for mRNA Vaccine Development

**DOI:** 10.3389/fimmu.2022.773264

**Published:** 2022-02-02

**Authors:** Han Lin, Kun Wang, Yuxin Xiong, Liting Zhou, Yong Yang, Shanwei Chen, Peihong Xu, Yujun Zhou, Rui Mao, Guangzhao Lv, Peng Wang, Dong Zhou

**Affiliations:** ^1^Department of Neurosurgery, Guangdong Provincial People’s Hospital, Guangdong Academy of Medical Sciences, Guangzhou, China; ^2^Department of Head and Neck Surgery, Cancer Hospital of Shantou University Medical College, Shantou, China; ^3^Department of Neurosurgery, The First Affiliated Hospital, Jinan University, Guangzhou, China; ^4^Division of Vascular Intervention Radiology, The Third Affiliated Hospital of Sun Yet-Sen University, Guangzhou, China; ^5^International Department, Affiliated High School of South China Normal University, Guangzhou, China; ^6^Shantou University Medical College, Shantou, China; ^7^Southern Medical University, Guangzhou, China; ^8^School of Medicine, South China University of Technology, Guangzhou, China

**Keywords:** mRNA vaccine, glioblastoma, tumor immune microenvironment, immunotyping, WGCNA, landscape

## Abstract

The use of vaccines for cancer therapy is a promising immunotherapeutic strategy that has been shown to be effective against various cancers. Vaccines directly target tumors but their efficacy against glioblastoma multiforme (GBM) remains unclear. Immunotyping that classifies tumor samples is considered to be a biomarker for immunotherapy. This study aimed to identify potential GBM antigens suitable for vaccine development and develop a tool to predict the response of GBM patients to vaccination based on the immunotype. Gene Expression Profiling Interactive Analysis (GEPIA) was applied to evaluate the expression profile of GBM antigens and their influence on clinical prognosis, while the cBioPortal program was utilized to integrate and analyze genetic alterations. The correlation between antigens and antigen processing cells was assessed using TIMER. RNA-seq data of GBM samples and their corresponding clinical data were downloaded from the Cancer Genome Atlas (TCGA) and the Chinese Glioma Genome Atlas (CGGA) for further clustering analysis. Six overexpressed and mutated tumor antigens (ARHGAP9, ARHGAP30, CLEC7A, MAN2B1, ARPC1B and PLB1) were highly correlated with the survival rate of GBM patients and the infiltration of antigen presenting cells in GBMs. With distinct cellular and molecular characteristics, three immune subtypes (IS1-IS3) of GBMs were identified and GBMs from IS3 subtype were more likely to benefit from vaccination. Through graph learning-based dimensional reduction, immune landscape was depicted and revealed the existence of heterogeneity among individual GBM patients. Finally, WGCNA can identify potential vaccination biomarkers by clustering immune related genes. In summary, the six tumor antigens are potential targets for developing anti-GBMs mRNA vaccine, and the immunotypes can be used for evaluating vaccination response.

## Introduction

Gliomas are intrinsic brain tumors arising from glial or precursor cells. They are classified into grades I to grade IV based on the degree of undifferentiation, anaplasia, and aggressiveness (1). Glioblastoma multiforme (GBM, grade IV glioma) accounts for 82% of all malignant gliomas and is characterized histologically by considerable vascular proliferation, cellularity and mitotic activity, and necrosis (2). Despite the availability of standard treatment options for GBM including surgical resection, radiotherapy and chemotherapy, the median survival time of GBM patients is only 12–15 months after diagnosis (3, 4). There has been growing evidence supporting the dynamic interaction between the central nervous system (CNS) and the systemic immune system. As a result, several studies have explored the efficacy of immunotherapy in the treatment of glioblastoma (5).

Currently, immunotherapies for gliomas include chimeric antigen receptor T cell therapy (CAR-T), immune-checkpoint inhibitors, oncolytic viral therapies, and vaccines (6). Cancer vaccines are classified into three major categories based on content and format, such as cell vaccines (tumor or immune cells), nucleic acid vaccines (viral vector, RNA or DNA), protein/peptide vaccines (7). Clinical trials evaluating the efficacy of immune cells (Dendritic cells e.g.), pulsed with TAAs (tumor-associated antigens) have revealed promising results. This is despite the heterogeneity in dose, route, and location of administration as well as the adjuvant used among different trials. HSP (Heat Shock Proteins) vaccines are a subclass of protein vaccines that are composed of HSPs bound to tumor peptides. Phase I HSP vaccine trial in patients with recurrent GBM appeared safe and tolerable (8). Several peptide vaccine trials targeting EGFRvIII showed that these peptide vaccines had a degree of effect with no significant toxicities encountered (9). Recently, a peptide vaccine targeting mutant IDH1 in newly diagnosed glioma has been evaluated in clinical trials and found to be safe, immunogenic, and efficacious (10). However, these vaccines still face with many challenges including: potential of tumor antigens escape, limited repertoire of using defined antigens, and high variability in the physicochemical properties (11, 12). Nucleic acid vaccines are a promising alternative that allow protein and peptide antigen to be expressed with the correct protein modifications in cells (13, 14).

DNA and mRNA vaccines are types of nucleic acid vaccines. The mRNA vaccines have several advantages over the DNA vaccines in safety and efficacy since mRNA does not need to enter the nucleus and be incorporated into the genome (15). Recently, there have been several studies evaluating the efficacy of mRNA vaccines in different types of tumors with varied outcomes being achieved. In gastrointestinal cancer, mutation-specific T cell responses were elicited against predicted neoepitopes and T cell receptors targeting KRAS^G12D^ mutation could be isolated after mRNA vaccines application (16). Two mRNA vaccines (CV-9103 and CV-9104) based on four prostate-specific antigens (STEAP, PSCA, PSMA, and PSA), have showed good tolerability and favorable immune-activation in phase I/II clinical trials in prostate cancer patients (17). Another mRNA vaccine targeting Trp2 induced antigen-specific T cell response and suppressed melanoma proliferation in preclinical trials (18). Angelique et al. reported that dendritic cells (DCs) transfected with CD133 mRNA (cancer stem cell marker) activated T cells, produced an effective and long-lived immune response, and suppressed the proliferation of CD133^+^ glioma stem cells (GSCs) and tumor growth in mice (19). Although there are very few studies that have evaluated the efficacy of mRNA vaccines in GBM, vaccination for GBMs TAA remains a viable concept and current trials are under way for several other targets.

The purpose of our study was to identify novel antigens of GBM that can be used as targets for mRNA vaccine development. In our study, we analyzed fraction alteration and gene expression data of GBMs and identified six candidate genes associated with poor prognosis and robust stimulation of the infiltration of antigen-presenting cells (APC). In addition, due to tumor complex immune microenvironment (TIME) and tumor heterogeneity, some tumor patients may be more likely to benefit from mRNA vaccines (20). We then developed a tool to identify GBM patients who might be more suitable for vaccination. To achieve this, we carried out cluster analysis of immune related genes, and identified three immune subtypes with distinct clinical, cellular, and molecular characteristics. The results were validated using an independent cohort. Finally, we used the immune landscape and immune gene co-expression modules to analyze the distribution of immune related gene characteristics in GBMs. The employed screening workflow was depicted in **Supplementary Figure 1**.

## Method and Material

### Identification of Tumor Associated Antigens of Glioblastoma Multiforme (GBM) for Vaccination

Cancer cells harbor unique mutant genes that theoretically create corresponding unique tumor-specific antigens (21). Besides, CNV (copy number variation) burden play significant role in tumors’ recurrence and death, indicating that CNV should be considered to be an antigen factor (22). With a deeper understanding of the immune system, the abberant expression of some gene products by tumor cells can be used to develop a variety of antigen-specific vaccination strategies and activate tumor antigen-specific T cells (23). Therefore, we analyzed the gene expression levels and gene alteration status in GBMs to identify tumor associated antigens. Gene Expression Profiling Interactive Analysis is a web-based tool used to process and deliver gene expression profiles based on the samples from GTEx (Genotype-Tissue Expression) and TCGA (the Cancer Genome Atlas) (24). In this study, GEPIA2 (http://gepia2.cancer-pku.cn) was employed to detect the differentially expressed genes between normal brain tissue and GBM (|Log2FC|>1, q<0.05) and explore their prognostic value in GBMs. Then, we applied the cBioPortal (http://www.cbioportal.org) for exploring, visualizing, and analyzing the genome alteration status of GBM antigens with multidimensional cancer genomics data (25). Last but not least, effectively uptake, processing, and presentation of antigens by APCs (antigen-presenting cells) can initiate antitumor immune responses through the activation of both CD4^+^ helper and CD8^+^ cytotoxic T lymphocytes (26–28). Tumor Immune Estimation Resource (TIMER, https://cistrome.shinyapps.io/timer/) can be used for systematically evaluating the clinical impact of different immune cells (dendritic cell, macrophage, neutrophil, CD8^+^ T cell, CD4^+^ T cell and B cell) in the tumor microenvironment of different tumor types. Therefore, we utilized TIMER to explore the relationship between expression of the identified potent antigens in GBMs and the degree of antigen-presenting cell (APC) infiltration. Spearman correlation analysis between APC cells and tumor purity was calculated using TIMER (29).

### Data Preprocessing

From The Cancer Genome Atlas (TCGA, https://portal.gdc.cancer.gov/), we downloaded FPKM RNA-seq dataset and mutect2-processed mutation dataset of 167 GBMs as well as their corresponding clinicopathological information. In addition, the RNA-seq data of 369 GBM cases and their corresponding clinicopathological information were obtained from the mRNAseq_325 and mRNAseq_693 datasets of the Chinese Glioma Genome Atlas (CGGA, http://www.cgga.org.cn/) and used as the validation dataset. The gene expression levels of the GBM samples were transformed using log2 for further analysis.

### Identification of Immune Subtypes and Their Cellular Characteristics

Patient stratification based on tumor immune subtypes can be used to distinguish patients who are suitable for vaccination therapy. After preprocessing the expression data of immune-related genes, we carried out consensus clustering to stratify GBM samples using the “ConsensusClusterPlus” package in R (30). Consensus Cumulative Distribution Function (CDF) was calculated to determine the optimal cluster number. Since the correlation between immune-related signatures and cancer markers is useful for tumor prognosis, we assessed the correlation between immune subtypes and 68 signatures from Wolf et al. (31). We then evaluated 28 immune cell signatures and LM22 signatures to assess the abundance of immune cells in each GBM tumor sample and compare the results among immune subtypes (32, 33). “ESTIMATE” package was employed to estimate Tumor purity scores (tumor purity, stromal score, immune score and estimate score) (34).

### Immune Landscape Analysis

Dimension reduction analysis is the commonly used analysis method to increase the understanding of biological systems in tumor. It maps high-dimensional space to low-dimensional space through orthogonal transformation and extracts the largest amount of data information by preserving the a few components (35). In the present study, dimension reduction analysis was conducted using the “monocele” package in R with Gaussian distribution (36, 37). Subsequently, the immune landscape was visualized using a functional map cell trajectory where different immune subtypes were identified by different colors.

### Gene Co-Expression Network

We constructed a scale-free network using the “blockwiseModules” function of the WGCNA package in R. The soft threshold of adjacency matrix was set as a continuous value between 0 and 1 so that the constructed network can be closer to the state of the real biological network (38). The “clusterProfile” package in R is a universal enrichment tool which integrates statistical analysis and visualization of functional profiles for genes and gene clusters (39). Then, the GO and KEGG functional components in the “clusterprofile” package were employed to analyze the biological function and pathways of modules associated with GBM prognosis.

### Statistical Analysis

The Wilcox test was used to compare data between the two groups, while the Kruskal Wallis test was used to compare three or more groups. Kaplan–Meier curves were used for OS analysis. The cut-off value was set as the best cut-off value from the “survminer” package in R. P<0.05 was regarded as statistically significant.

## Result

### Exploring Potential Tumor Antigens of Glioblastoma Multiform (GBM)

**Figure 1:**
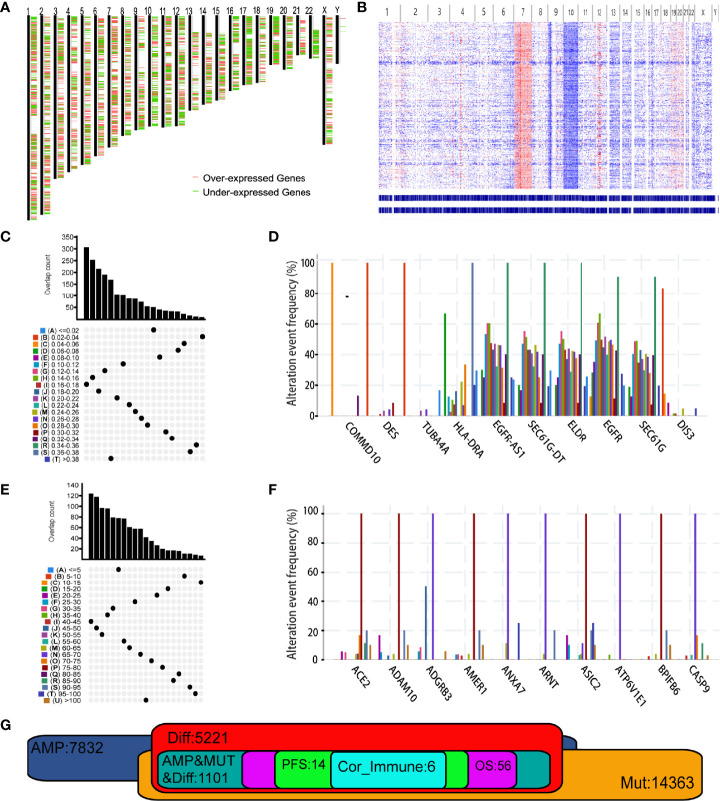
Identification of potential tumor antigens of Glioblastoma multiforme (GBM). **(A)** Chromosomal distribution of up- and down-regulated genes in GBMs. Red plot: overexpressed genes; Green plot: under-expressed genes. **(B)** The chromosomal distribution of the aberrant copy number genes in GBMs. Red plot: amplified genes; Blue plot: deleted genes. **(C)** Samples overlapping in altered genome fraction groups. **(D)** Top ten genes with highest frequency in altered genome fraction. **(E)** Samples overlapping in mutation count groups. **(F)** Top ten genes with highest frequency in mutation count groups. **(G)** Potential tumor antigens (total 1106) with overexpression, mutation, and amplification in GBM, and significant association with OS, RFS and immune infiltration (total 6 candidates).

At the beginning, results of GEPIA2 analysis revealed 7664 differentially expressed genes where 5221 genes were overexpressed in GBM when compared to normal brain tissue (**Figure 1A**). Using the cbioportal analysis algorithm, we identified 7832 amplified genes as potential tumor antigens of GBM (**Figure 1B**). In the fraction genome altered group, ten genes with the highest alteration frequency included COMMD10, DES, TUBA4A, HLA-DRA, EGFR-AS1, SEC61G-DT, ELDR, EGFR, SEC61G and DIS3 (**Figures 1C, D**). Moreover, a total of 14363 mutated genes were identified by analyzing their mutation count in GBMs (**Figure 1E**). Among them, we displayed ten genes with the highest mutation frequency in GBM, including ACE2, ADAM10, ADGRB3, AMER1, ANXA7, ARNT, ASIC2, ATPGV1E1, BPIFB6 and CASP9 (**Figure 1F**). Overall, 1101 amplified, mutated, and overexpressed genes were identified for further analysis (**Figure 1G**).

### Identification of Tumor Antigens Associated With GBM Prognosis and Antigen Presenting Cells

**Figure 2:**
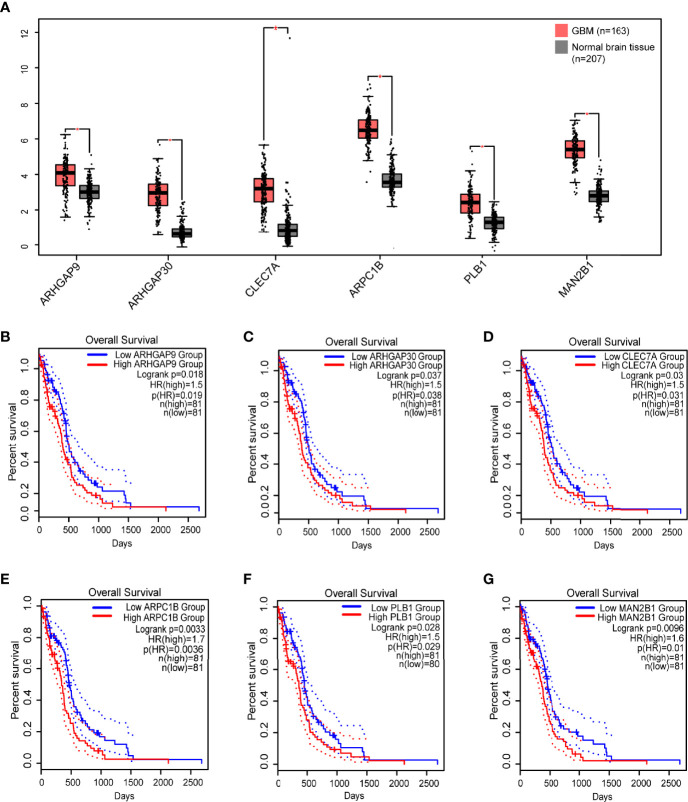
Identification of tumor antigens associated with GBM prognosis. **(A)** Differential expression of six candidates in normal brain tissue and GBMs. *, significant difference. Kaplan-Meier curves showing OS of GBM patients stratified on the basis of **(B)** ARHGAP9, **(C)** ARHGAP30, **(D)** CLEC7A, **(E)** ARPC1B, **(F)** PLB1 and **(G)** MAN2B1 expression levels. 50% (Median) cutoff was set up for dividing low and high expression groups. Log-rank test was used for hypothesis testing, and a *p*-value <0.05 was considered statistically significant.

**Figure 3:**
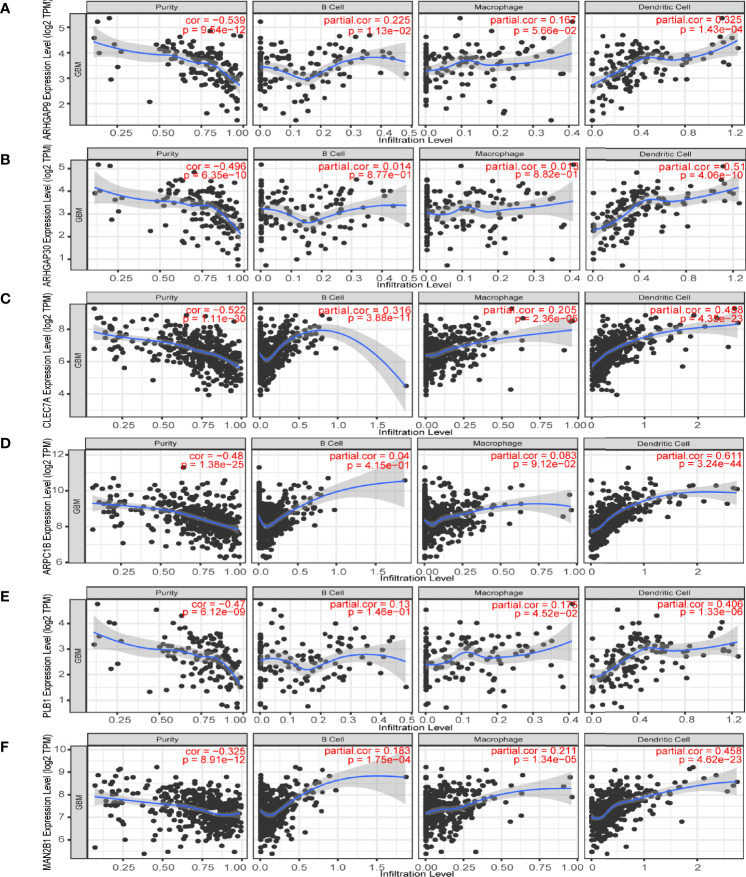
Identification of tumor antigens associated with antigen-presenting cells (APCs). Correlation between the expression levels of **(A)** ARHGAP9, **(B)** ARHGAP30, **(C)** CLEC7A, **(D)** ARPC1B, **(E)** PLB1 and **(F)** MAN2B1 and infiltration of APCs (macrophages, dendritic cells, and B cells) in GBM.

Then, we analyzed the role of 1101 aforementioned genes in the survival and immune response of GBM patients. Results of survival analysis showed that the expression of 14 genes could predict OS (Overall Survival) and PFS (Disease Free Survival) of GBM patients (**Figure 2** and **Supplementary Figure 2**). More importantly, the expression levels of six genes (ARHGAP9, ARHGAP30, CLEC7A, MAN2B1, ARPC1B, and PLB1) were positively correlated with the level of abundance of B cells, macrophages, and dendritic cells (DCs) and were thus considered suitable targets for vaccine use (**Figure 3**). In general, their upregulation was related to poorer GBM prognosis and more APCs infiltration. Hence, these tumor antigens play key roles in the development and progression of GBM and could be directly processed and presented by the APCs to T cells or recognized by the B cells to trigger an immune response.

### Immune Subtypes of GBM

**Figure 4:**
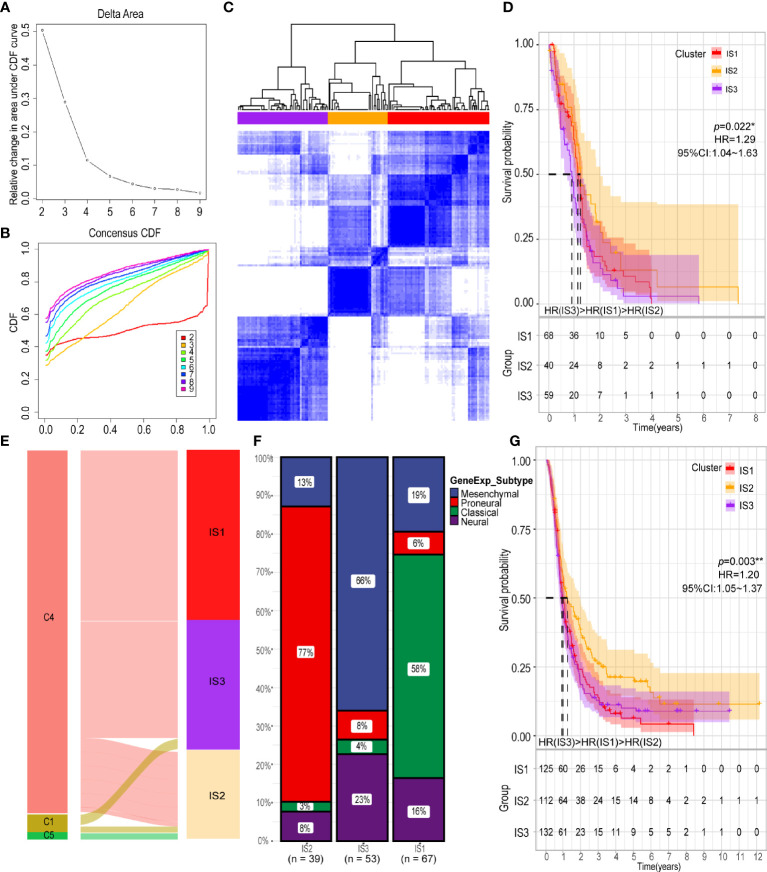
Identification of potential immune subtypes of GBM. **(A)** Cumulative distribution function curve and **(B)** delta area of immune-related genes in TCGA cohort. **(C)** Sample clustering heat map. **(D)** Kaplan-Meier curves showing OS of GBM immune subtypes in TCGA cohort. **(E)** Overlap of GBM immune subtypes with three pan-cancer immune subtypes (NC1 = 2, NC4 = 158, NC5 = 1). **(F)** Distribution of four gene expression subtypes across IS1-IS3 in TCGA cohort. **(G)** Kaplan-Meier curves showing OS of GBM immune subtypes in CGGA cohort. *p < 0.05, **p < 0.01.

Next, we analyzed the immune status of GBM and identified patients likely to benefit from vaccination by conducting immunotyping. With expression profiles of 1658 immune related genes, we constructed consensus clustering of 167 GBMs from TCGA cohort and validated the stratification using GBM samples from the CGGA cohort. Due to the relatively small sample size of discovery cohort, we chose k = 3 to group the samples into three immune subtypes (IS) (**Figures 4A–C**). The GBM patients in the IS2 group had the best survival prognosis, followed by GBM patients in the IS1 group; GBM patients in the IS3 group had the worst prognosis (**Figure 4D**). We then explore the relationship between the six pan-cancer immune subtypes (C1-C6) and the three immune subtypes identified in this study (40). The immune subtypes identified in this study mainly clustered into C1, C4 and C5 pan-cancer immune subtypes (**Figure 4E**). With differential response to radiotherapy and chemotherapy, four GBM subtypes (proneural, neural, mesenchymal, and classical) were identified before (41–43). Among them, GBMs from IS2 were overlapped by proneural subclass, those from IS3 were covered by mesenchymal subclass and GBMs from IS1 were mainly overlapped by classical subclasses (**Figure 4F**) The immune subtypes in the CGGA cohort were associated with prognosis, which was consistent with the results obtained from the TCGA cohort (**Figure 4G**). Findings from our study suggest that our classification of GBMs might be more detailed than the classification according to the pan-cancer immune subtypes. Thus, our classification provides more useful guideline for development and execution of the GBM immunotherapy strategy.

### The Association Between Tumor Mutational Burden (TMB) and Immune Subtypes

Tumor mutation burden is significant for the efficacy of vaccines since the abundance of antigens and neoantigens affects the immunogenicity of the tumor (44). We assessed the TMB and mutations data of the GBMs from the TCGA cohort based on the three subtypes. Ten most frequently mutated immune-related genes (EGFR, PIK3CA, PIK3R1, PIK3CG, ANK1, PDGFRA, SEMA3C, TG, TMPRSS6 and L1CAM) was showed with waterfall plot (**Supplementary Figure 3A**). We found the number of mutated genes and the tumor mutational burden was significant different among three subtypes (**Supplementary Figures 3B, C**). These findings indicate that the three immune subtypes expressed distinct amounts of tumor antigens from mutated genes and that the IS3 subtype had the least immunogenicity among these subtypes.

### The Association Between TIME and the Immune Subtypes

**Figure 5:**
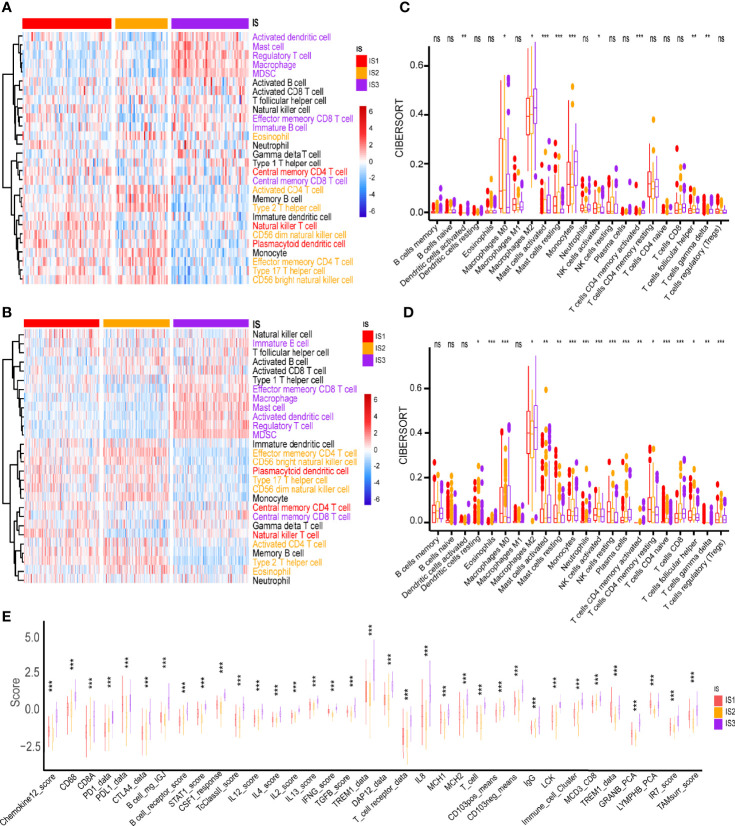
Cellular and molecular characteristics of immune subtypes. Differential enrichment scores of 28 immune cell signatures among GBM immune subtypes in **(A)** TCGA and **(B)** CGGA cohorts. Different colors of the text on the right represents the immune cells were more enriched in the corresponding subtypes. Differential enrichment scores of CIBERSORT 22 immune cell signatures in **(C)** TCGA and **(D)** CGGA cohorts. **(E)** Differential enrichment scores of 56 immune signatures among GBM immune subtypes. *p < 0.05, **p < 0.01, ***p < 0.001. ns, no significance.

**Figure 6:**
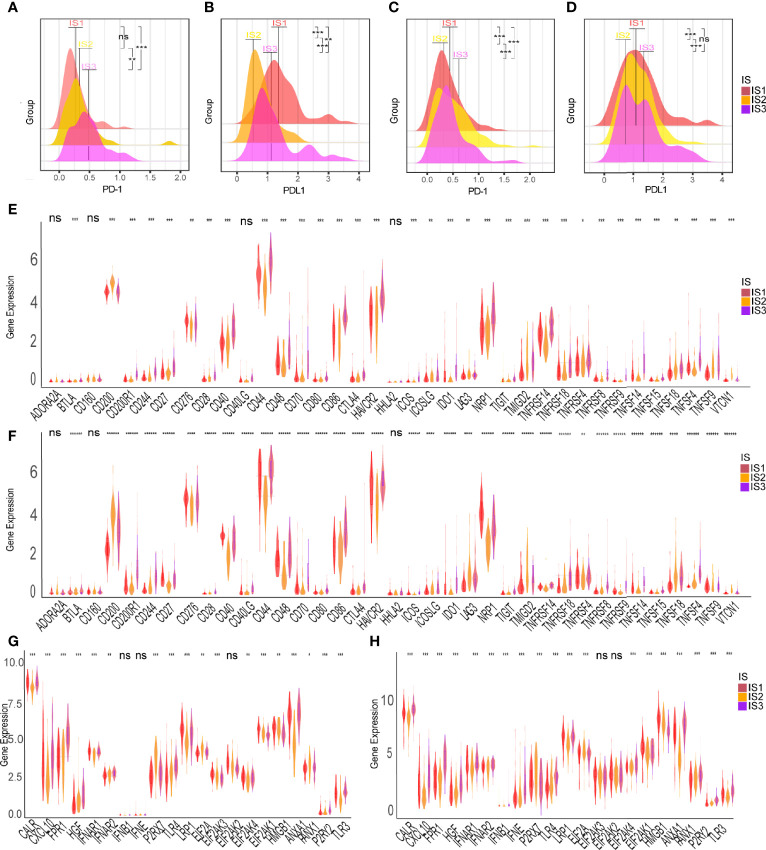
Association between immune subtypes and ICPs and ICD modulators. Differential expression of PD-1 among the GBM immune subtypes in **(A)** TCGA and **(C)** CGGA cohorts. Differential expression of PD-L1 among the GBM immune subtypes in **(B)** TCGA and **(D)** CGGA cohorts. Differential expression of ICP genes among the GBM immune subtypes in **(E)** TCGA and **(F)** CGGA cohorts. Differential expression of ICD modulator genes among the GBM immune subtypes in **(G)** TCGA and **(H)** CGGA cohorts. *p < 0.05, **p < 0.01, ***p < 0.001, ****p<0.0001, ******p<0.000001. ns, no significance.

The ssGSEA method was used to identify the 28 immune signatures previously reported in both TCGA and CGGA cohorts where immune subtypes showed different proportions of immune cell components (**Figures 5A, B**). For example, the scores of mast cells, MDSC, activated dendritic cells and macrophages were significantly higher in IS3; eosinophils, type 2 T helper cells and activated CD4 T cells were higher in IS2; while central memory CD4 T cells, plasmacytoid dendritic cells and NK T cells were more abundant in IS1. Analysis of CIBERSORT algorithm showed that there were more immunosuppressive regulatory immune cells (macrophage M2 and gamma delta T cells) but less cytotoxic immune cells (activated NK cells) in the IS3 subtype compared to the IS1 and IS2 subtypes (**Figure 5C**). Thus, the IS2 subtype can be considered to be immunologically “hot”, the IS1 to be in an intermediate state, and the IS3 subtype to be immunologically “cold”. A similar trend was seen in the CGGA cohort (**Figure 5D**). Besides, the expression of most immune signatures was higher in the IS3 subtype compared to the IS2 and IS1 subtypes (**Figure 5E**). Immune checkpoints (ICPs) (e.g., PD1 and PDL1) and immunogenic cell death modulators (e.g., HGF and IFN) are crucial for modulating the immune responses of effectors and maintaining the self-tolerance of tumor to minimize tissue damage (45, 46). PD1 and PDL1 are the most common ICPs for blockage therapy. The IS2 subtype had the least expression of PD1 and PDL1 among the three subtypes; meanwhile, IS3 has highest level of PD1 expression which suggested they might show good response rates for immune checkpoint blockade (**Figures 6A–D**). Subsequently, we assessed the differential expression of and ICD modulators and ICPs between the three immune subtypes. We found that 34 ICPs had distinct expression patterns among the three subtypes. There was significant upregulation of CD28, CD244, CD200R1, CD27, CD40, CD40LG, CTLA4, CD48, CD86, CD80, CD70, HAVCR2, ICOS, ICOSLG, IDO1, NRP1, TIGIT, TNFRSF14, TNFRSF18, TNFRSF8, TNFRSF9, TNFRSF14, TNFSF4, and TNFSF9 in the IS3 subtype (**Figures 6E, F**). In addition, almost all ICDs were differentially expressed among the three subtypes (**Figures 6G, H**). Taken together, immunotyping can be considered a biomarker of immune status in GBMs and can be used to predict the response of the patients to vaccination.

### The Immune Landscape of GBM

**Figure 7:**
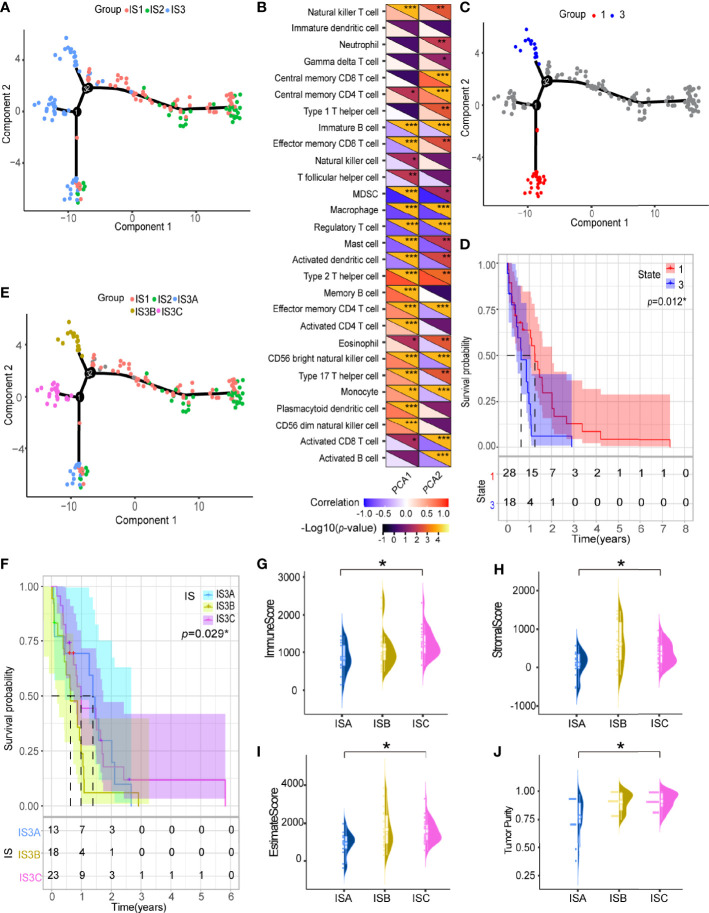
Immune landscape of GBM. **(A)** Immune landscape of GBMs where each point represents a patient, and the immune subtypes are color-coded. The horizontal axis represents the first principal component, and the vertical axis represents the second principal component. **(B)** Heat map of two principal components with 28 immune cell signatures. Immune landscape of samples from **(C)** two extreme locations and **(D)** their prognostic status. **(E)** Immune landscape of the subsets of GBM immune subtypes. **(F)** Different subsets in IS3 associated with different prognoses. Immune assessment of different subsets in IS3, represented by **(G)** immune score, **(H)** stromal score, **(I)** estimate score and **(J)** tumor purity. *p < 0.05, **p < 0.01, ***p < 0.001.

We further explored the immune characteristics of GBM by integrating the immune-related gene expression profiles from TCGA cohort to construct the immune landscape where the immune distribution of each GBM were visualized (**Figure 7A**). The expression profiles were aggregated and visualized in a two-dimensional scatter plot with several branches using the DDRTree algorithm (a manifold learning approach) after dimensionality reduction. Component 1 was highly correlated with natural killer cells, activated CD4 T cells and type 17 T helper cells, while component 2 was positively correlated with central memory CD8 T cells, central memory CD4 T cells, type 1 T helper cells, and natural killer cells (**Figure 7B**). Further prognostic analysis of the GBMs distributed on the extreme ends of the branches showed that patients in group 3 had poorer prognosis than those in group 1, indicating that the immune landscape can be used to discriminate the patients and predict their prognosis (**Figures 7C, D**). The GBMs of the IS3 subtypes were further stratified into three immune subtypes, IS3A, IS3B and IS3C, based on the distribution of the individual GBM samples in the immune landscape (**Figure 7E**). Among them, IS3A had better prognosis than the other types (**Figure 7F**). The estimate score and expression of ICP and ICD were significantly different among these immune subtypes (**Figures 7G–J** and **Supplementary Figures 4A, B**). Interestingly, more activated B cells, cytotoxic T cells and NK cells were located in IS3A than that of IS3B and IS3C, indicating the GBMs of IS3A showed more inflamed microenvironment (**Supplementary Figure 4C**). These findings suggest that the immune landscape is an important complement to immunotyping.

### WGCNA Analysis of Immune Related Genes in GBM

**Figure 8:**
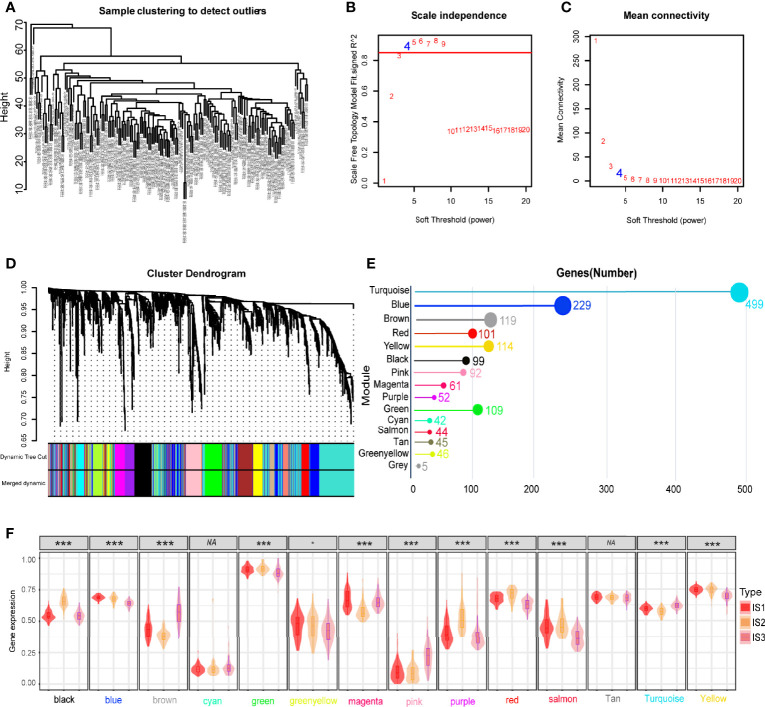
Identification of immune gene co-expression modules of GBM. **(A)** Sample clustering. **(B)** Scale-free fit index for various soft-thresholding powers (β). **(C)** Mean connectivity for various soft-thresholding powers **(D)** Dendrogram of all differentially expressed genes clustered based on a dissimilarity measure (1-TOM). **(E)** Gene numbers in each module. **(F)** Differential distribution of feature vectors of each module in GBM subtypes. *p < 0.05, **p < 0.01, ***p < 0.001.

**Figure 9:**
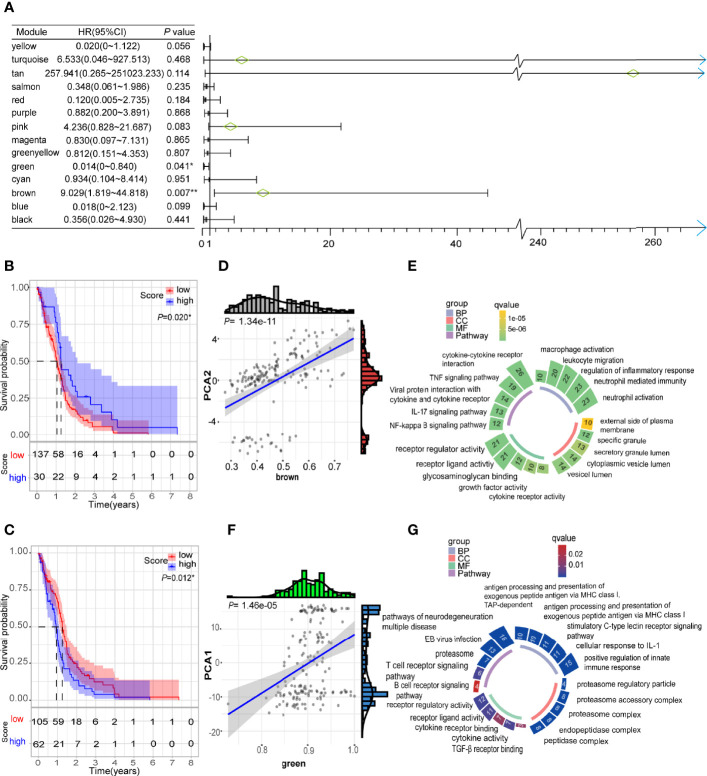
Identification of immune hub genes of GBM. **(A)** Forest maps of single factor survival analysis of 14 modules of GBM. **(B)** Differential prognosis in brown module with high and low mean. **(C)** Differential prognosis in green module with high and low mean. **(D)** Correlation between brown module feature vector and second principal component in immune landscape. **(E)** Circular barplot showing GO term (BP: biology process; CC: cellular component; MF: molecular function) and KEGG term (pathway) in the brown module. The barplot size and color intensity represent the gene count and enrichment level respectively. **(F)** Correlation between green module feature vector and first principal component in immune landscape. **(G)** Circular barplot showing GO term (BP, biology process; CC, cellular component; MF, molecular function) and KEGG term (pathway) in the green module. The barplot size and color intensity represent the gene count and enrichment level respectively. *p < 0.05.

Weighted gene co-expression network analysis (WGCNA) was employed to conduct co-expression analysis of the identified immune related genes, and the results were visualized using a dendrogram (**Figure 8A**). In the scale-free network, the optimal soft threshold was set at 4 (**Figures 8B, C**). The colors of the dendrogram branches indicate different gene clusters (min module size = 20, deep split = 4 and height = 0.25) (**Figure 8D**). In the end, 13 gene modules were clustered except for the grey module (**Figure 8E**). The module eigengenes of IS2 were more abundant in the black, green, red, yellow, and purple modules, while the brown, pink and turquoise modules had more eigengenes of IS3 (**Figure 8F**). Moreover, this study revealed that the module eigengenes of the green and brown modules were significantly associated with the prognosis of GBMs (**Figures 9A–C**). Interestingly, the modules eigengenes of brown and green modules were positively correlated with component 2 and component 1, respectively (**Figures 9D, F**). Biological functions analysis of the prognosis-related modules showed that genes of the brown module were enriched in macrophage activation, IL-17 signaling pathway and cytokine-cytokine receptor interaction (**Figure 9E**). The genes in the green module were enriched in antigen processing, B cell receptor signaling pathway and T cell receptor signaling pathway (**Figure 9G**). In the end, thirteen genes including PLAUR, F13A1, THBD, CD300E, HK3, FPR2, SOCS3, NDUFB9, PSMD6, PSMD10, CACYBP, GEMIN6 and PSMD14 were identified as hub genes with >80% relevance in the brown and green modules. These genes were considered to be potential biomarkers for the mRNA vaccine.

## Discussion

Glioblastoma multiforme (GBM) is characterized by high degree of malignancy and poor prognosis. The standard treatment plan for GBM includes maximal safe resection, radiotherapy, and concurrent administration of temozolomide (TMZ) (1). However, the therapeutic outcomes for these strategies are poor with a median OS of just 12-14 months (47). There is therefore need for novel and improved therapeutic alternatives for GBM. Cancer vaccines and other immunotherapies (CAR-T, ICP blockage and so on) are promising alternative strategies for the treatment of GBMs, although they are influenced by immune escape or immunosuppression of the microenvironment (48). Tumor associated antigens are antigens preferentially expressed in cancer cells which arise due to somatic mutations or growth-related factors (49). These antigens are good candidates for cancer vaccine development. The detection and analysis of these antigens can quickly lead to the identification of suitable human mRNA vaccine targets. Recently, analysis of potential tumor antigen in pancreatic adenocarcinoma and cholangiocarcinoma provided novel insights for mRNA vaccine development of these tumors (50, 51). However, there have been no comprehensive analysis of potential GBM antigens that can be used for the development of an anti-GBM vaccine.

In this study, we identified six tumor antigens (ARHGAP9, ARHGAP30, CLEC7A, MAN2B1, ARPC1B and PLB1) that had mutations, amplification and were overexpressed in GBMs, as promising candidates for mRNA vaccine. ARHGAP30 (Rho GTPase activating protein 30) and ARHGAP9 (Rho GTPase activating protein 9) are proteins containing a Rho-GTPase activating (Rho-GAP) domain. These proteins are critical in the modulation of several tumorigenic pathways (p38/MAPK, FOX, Wnt/β-caterin pathways) and are significantly associated with the prognosis of breast tumor, bladder carcinoma and other cancers (52–56). CELC7A is a pattern recognition receptor that detects glucan-like structures to trigger the phagocytic activity of macrophages (57). CELC7A (C-type lectin domain family 7, member A) ligates galectin-9 in the tumor microenvironment of pancreatic ductal adenocarcinoma, resulting in the suppression of T cell immunogenicity and reprogramming of tolerogenic macrophages (58). ARPC1B is a constituent of the actin-related protein 2/3 (ARP2/3) complex which binds and activates Aurora A to regulate centrosome integrity (59). Since ARPC1B is required for actin reorganization and lamellipodia formation, ARPC1B mutations induce dysfunction of cytotoxic T cells (60). There are several reports indicating that mutant ARPC1B plays a significant role in immunodeficiency diseases (61–63). It was reported mutations of rs117512489 in PLB1 (phospholipase B1) was associated with the prognosis of the patients with non-small cell lung cancer (64). Results of next generation sequencing validated the role of PLB1 as a biomarker for lung cancers (65, 66). In the present study, the expression of these six antigens was positively associated with the abundance of antigen presenting cells (APCs), suggesting that they might have potential as vaccine targets and stimulate APC activation (67, 68). However, there is need for further research to determine their mechanisms of action against tumors.

In our study, we analyzed the characteristics of GBMs based on the pan-cancer immune categories and found that a majority of the GBMs had characteristics corresponding to the C4 (Lymphocytes Depleted) category. A few GBMs had characteristics corresponding to the C1 (Wound Healing) and C5 (Immunologically Quiet) categories. In fact, only a small population of tumor patients responds to the vaccine treatment, although there is a lack of tools to select patients likely to benefit from the treatment and evaluate immune response. Therefore, we clustered GBMs based on the expression of integrated immune related gene profiles, so as to provide a guideline for the application of anti-GBM mRNA vaccine. The GBM samples were clustered into three immune subtypes with different clinical prognosis and immune profiles. GBMs in the IS3 subtype had the poorest survival compared to the IS1 and IS2 subtypes, indicating that immunotyping is a potential prognostic biomarker for GBMs. More importantly, there may be distinct mechanisms involved in the modulation of the tumor immune environment among the three GBM subtypes, suggesting that different therapeutic strategies are required for each subtype. GBMs with IS1 and IS2 represent the lack of regulatory immune cells and immunosuppressive antigen-presenting cells, resulting in T cell activation and survival advantage. Therefore, the use of immunotherapy in these patients can induce a stronger immune response. On the contrary, the immune-cold subtype (IS3) had a highly complex and thornier tumor microenvironment. Generally, macrophages are recruited into the tumor in response to inflammation-mediated chemokines, where they engulf tumor cells and present antigens to adaptive immune cells (69). GBM cells can utilize paracrine metabolites or surface signals to polarizes these macrophages toward the anti-inflammatory M2 phenotype (70). These tamed M2 macrophages expressed immune checkpoint molecules to suppress adaptive immune anti-tumor response, that supports the survival of cancer stem cells (71). The high expression of ICP (PD-1, PD-L1, LAG-3, etc.), in the GBM samples of the IS3 subtype was an indication that there was severe suppression of lymphocyte activation but exhaustion of existing T cells (72–74). Furthermore, several immunosuppressive factors such as TGF-β and IL-10 released by GBM cells, Tregs, microglia, macrophages, also lead to local immunosuppression (75, 76). These results indicated that GBMs of the IS3 subtype were more likely to benefit from vaccination in combination with immune checkpoint blockage. In addition, the use of CAR T-cell therapy deserves to be explored.

Gliomas have poor clinical outcomes, due to the high degree of intratumoral heterogeneity (ITH) and low efficacy of immunomodulator (IM) therapy compared with other tumor types (77). Several studies indicated that the ITH of GBM contributes to the development of chemoradiotherapy resistance and different subpopulations of GBM respond differently to treatment, resulting in the generation of treatment-refractory recurrent tumors (78, 79). The machine learning-based analysis led to dimensionality reduction of the expression profile of GBMs, revealing the intra-cluster heterogeneity in GBMs. From this analysis, GBMs in the IS3 subtype were further subdivided into three subtypes. Among the three subtypes, IS3A showed significantly better survival than the other two subtypes. Estimate score analysis of the GBMs in the IS3 subtype reflected various immune factors while therapeutic estimation and application of combination therapy on these patients was required. It has been reported that combined immunotherapy involving the use of anti-CTLA-4 monoclonal antibody and vaccine can modulate the tumor microenvironment and enhance anti-tumor immune response compared to the vaccine or monoclonal antibody alone in triple negative breast cancer (80). Integrating clustering and immune landscape can provide more detailed immune profile of GBM and assist clinicians in designing accurate immunotherapy strategies.

Despite the previous reports on vaccine showed potential of GBM vaccination treatment, there is great limit of current peptide vaccines’ efficacy. For example, IDH mutation frequencies were less than 10% of primary GBM which influence the efficacy of IDH targeting vaccine in primary GBMs (81). As for EGFR*vIII* targeting vaccine, it was proven for safety but no benefit to overall survival because of immunoediting under immunologic pressure (82). With more flexibility, mRNA vaccine is equipped with several advantages: not require prior knowledge, not restricted by the patient’s HLA type (14). Recently, several clinical trials on mRNA vaccine therapy are ongoing (NCT02649582, NCT02808364 and NCT02709616). Interestingly, the results of a preclinical trial by Duane A showed that TMZ enhances vaccine-driven immune responses and significantly reduces GBM growth in a murine model, which suggested that vaccines therapy can act synergistically with chemotherapy in furthering a therapeutic effect (83). GBM patients who underwent vaccine therapy targeting CMV pp65 exhibited unexpectedly prolonged progression free survival (PFS) and overall survival (OS) (84). Notably, GBMs hampers immunotherapy with low infiltration of lymphocytes but high fractions of macrophages and there is need for further research on the anti-GBM mRNA vaccine and combined protocol (85). Our study comprehensively analyzed the potential antigens and the conditions for the application of the mRNA vaccine in GBMs. The results from this study provide a theoretical foundation for mRNA vaccine development and combined immunotherapy for GBMs.

## Conclusion

ARHGAP9, ARHGAP30, CLEC7A, MAN2B1, ARPC1B and PLB1 were identified as potential GBM antigens for mRNA vaccine development. GBM patients in the IS3 subtype are more likely to benefit from vaccination.

## Data Availability Statement

The datasets analyzed during the current study are available in The Cancer Genome Atlas database (TCGA, https://portal.gdc.cancer.gov/) and Chinese Glioma Genome Atlas (CGGA, http://www.cgga.org.cn/).

## Author Contributions

HL and KW designed the study, checked the data and prepared the manuscript. YX and LZ performed data collection. GL, YY, and RM searched the literature and took part in the manuscript preparation. PX, SC, and YZ conduct statistical analysis. PW and DZ supervised this project. All authors read and approved the final manuscript.

## Funding

This program was financially supported by Natural Science Foundation of China (NO.81901250), High-level Hospital Construction Project of Guangdong Province of China (NO. DFJH201924), GDPH Scientific Research Funds for Leading Medical Talents and Distinguished Young Scholars in Guangdong Province (NO. KJ012019434) and Natural Science Foundation of Guangdong Province of China (NO.2018A0303130236).

## Conflict of Interest

The authors declare that the research was conducted in the absence of any commercial or financial relationships that could be construed as a potential conflict of interest.

## Publisher’s Note

All claims expressed in this article are solely those of the authors and do not necessarily represent those of their affiliated organizations, or those of the publisher, the editors and the reviewers. Any product that may be evaluated in this article, or claim that may be made by its manufacturer, is not guaranteed or endorsed by the publisher.

## Supplementary Material

Supplementary Figure 1 Procedures for the analysis of antigen detection and GBM immune subtypes identification.

Supplementary Figure 2 Association between the expression of tumor antigens with GBM RFS. Kaplan-Meier curves showing PFS of GBM patients stratified on the basis of **(A)** ARHGAP9, **(B)** ARHGAP30, **(C)** CLEC7A, **(D)** ARPC1B, **(E)** PLB1 and **(F)** MAN2B1 expression level. p-value <0.05 was considered statistically significant.

Supplementary Figure 3 Association between immune subtypes and TMB and mutation. **(A)** Ten highly mutated genes in GBM immune subtypes. **(B)** mutation number and **(C)** TMB in GBM IS1-IS3. **p* < 0.05.

Supplementary Figure 4 Association between immune subtypes and immune molecules and immune cells signatures. Differential expression of **(A)** ICP genes and **(B)** ICD genes among the GBM immune subtypes. **(C)** Differential enrichment scores of 28 immune cell signatures in the above subsets. **p* < 0.05, ***p* < 0.01, ****p* < 0.001.
