# Supplementary material for: Identification of Tumor Antigens and Immune Subtypes of Glioblastoma for mRNA Vaccine Development
Source: Front Immunol. 2022 Feb 2;13:773264. doi: 10.3389/fimmu.2022.773264 (PMC8847306; doi:10.3389/fimmu.2022.773264)
Supplement: Supplementary Figure 1 — Procedures for the analysis of antigen detection and GBM immune subtypes identification. [file DataSheet_1.docx]

Supplementary Material


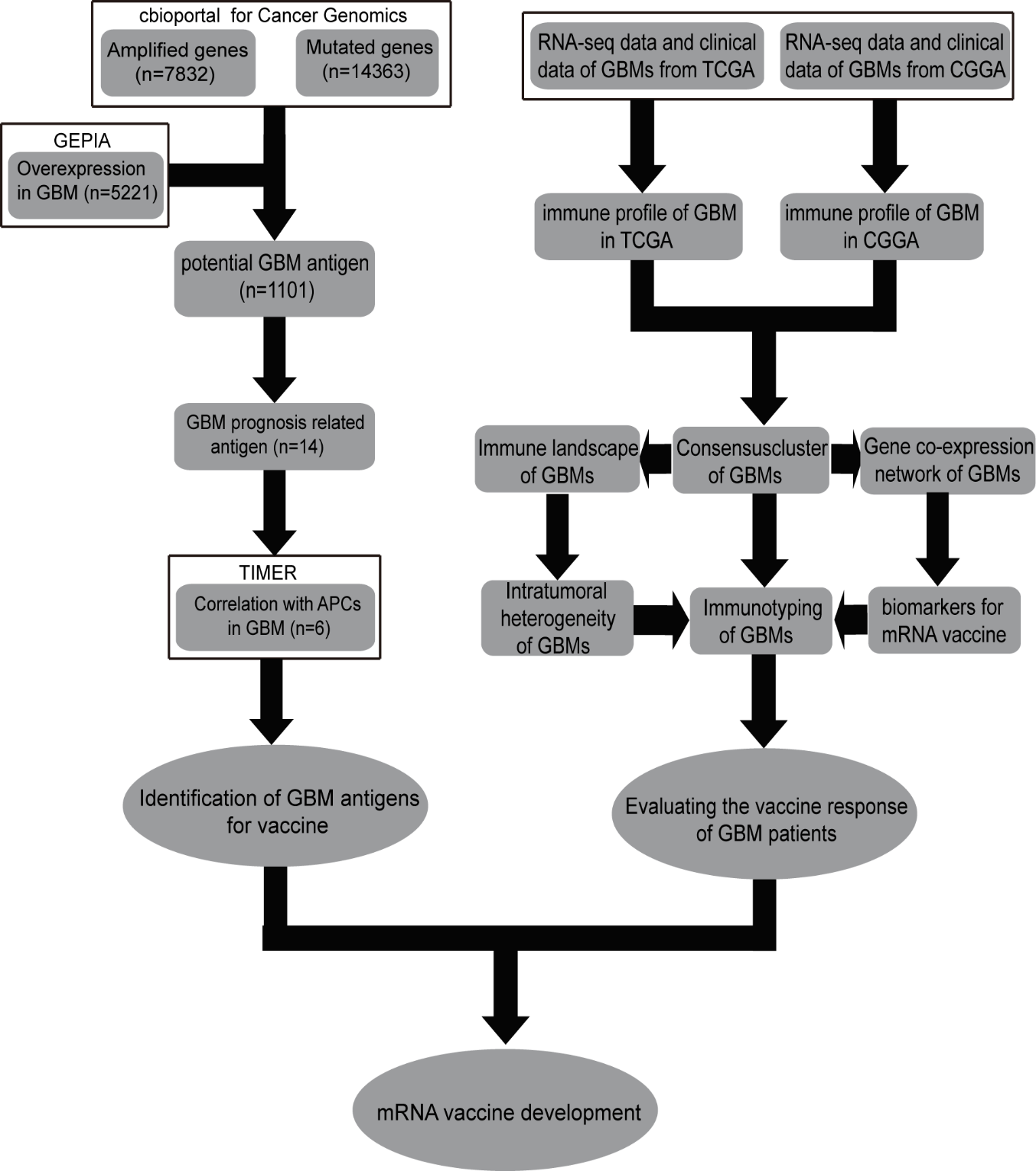


**Supplementary Figure 1.** Procedures for the analysis of antigen detection and GBM immune subtypes identification


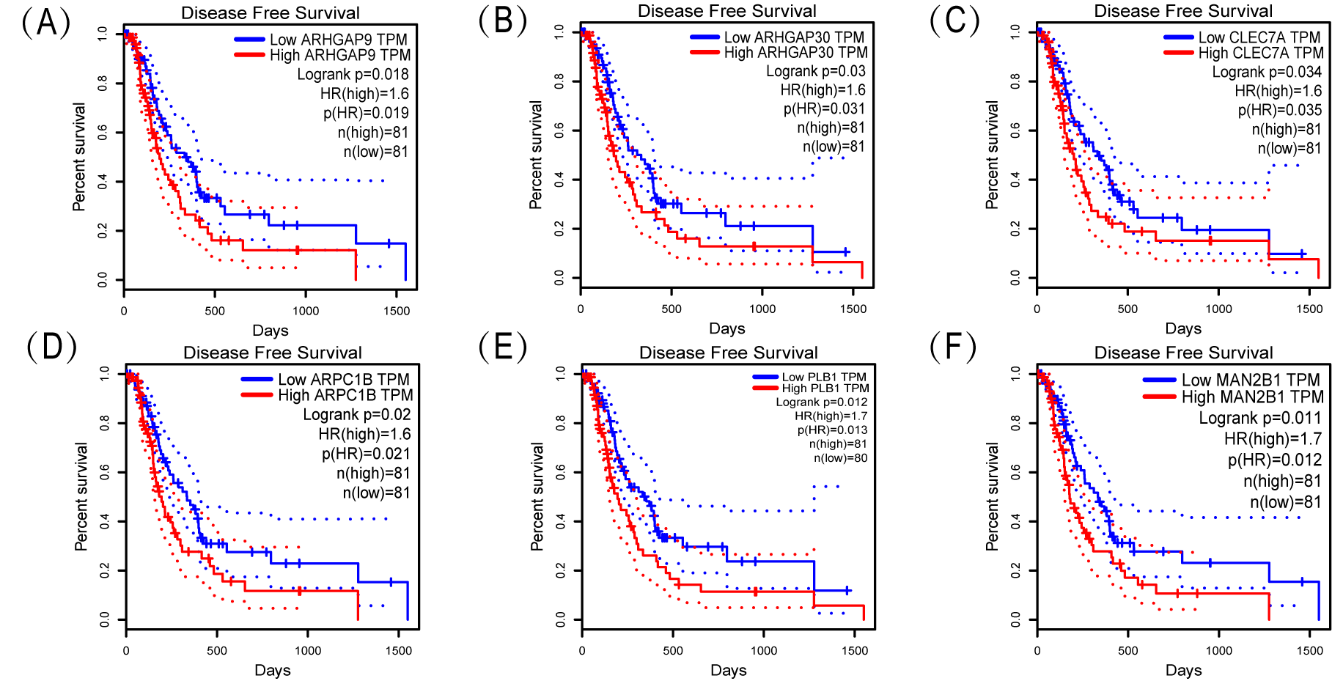
Supplementary Figure 2. Association between the expression of tumor antigens with GBM RFS. Kaplan-Meier curves showing PFS of GBM patients stratified on the basis of (A) ARHGAP9, (B) ARHGAP30, (C) CLEC7A, (D) ARPC1B, (E) PLB1 and (F) MAN2B1 expression level. p-value <0.05 was considered statistically significant.


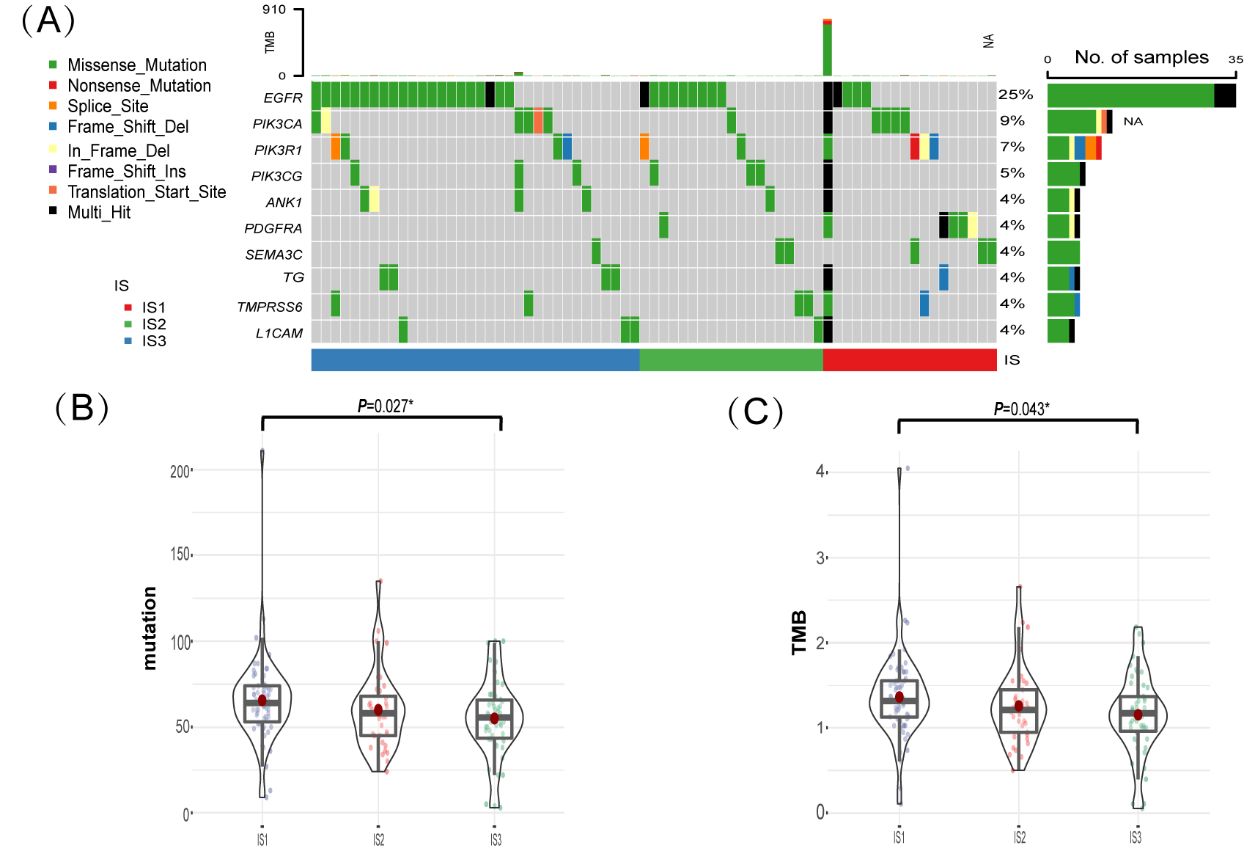


**Supplementary Figure 3.** Association between immune subtypes and TMB and mutation. (A) Ten highly mutated genes in GBM immune subtypes. (B) mutation number and (C) TMB in GBM IS1-IS3. * *p* < 0.05.


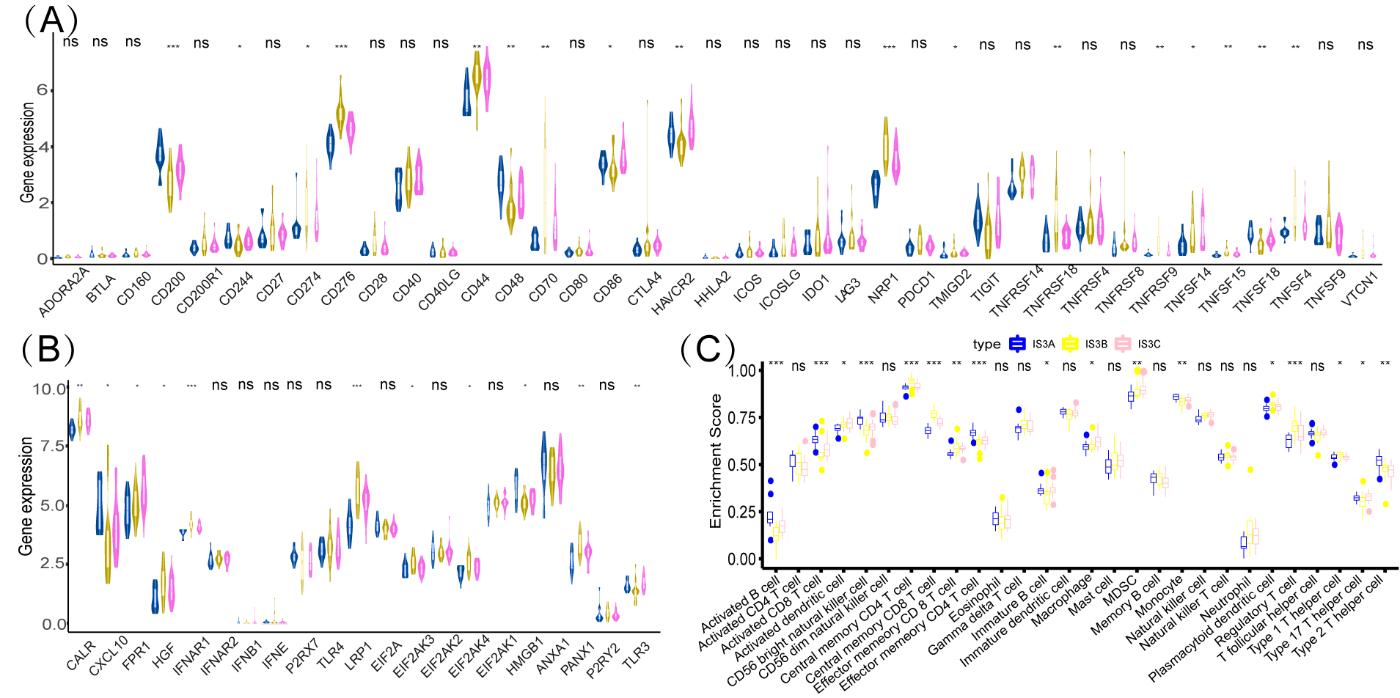


**Supplementary Figure 4.** Association between immune subtypes and immune molecules and immune cells signatures. Differential expression of (A) ICP genes and (B) ICD genes among the GBM immune subtypes. (C) Differential enrichment scores of 28 immune cell signatures in the above subsets. * *p* < 0.05, ** *p* < 0.01, *** *p* < 0.001.
